# Acute Hydrops with Total Corneal Edema in a Very Young Child with Keratoconus: The Youngest Age Reported Case

**DOI:** 10.1155/2022/2381703

**Published:** 2022-08-12

**Authors:** Mohammad-Reza Sedaghat, Hamed Momeni-Moghaddam, Michael W. Belin, Maryam Savardashtaki, Shehzad A. Naroo, Hassan Robabi

**Affiliations:** ^1^Eye Research Center, Mashhad University of Medical Sciences, Mashhad, Iran; ^2^Health Promotion Research Center, Zahedan University of Medical Sciences, Zahedan, Iran; ^3^University of Arizona, Department of Ophthalmology & Vision Science, Tucson, Arizona, USA; ^4^Department of Optometry, School of Paramedical Sciences, Mashhad University of Medical Sciences, Mashhad, Iran; ^5^College of Health and Life Sciences, Aston University, Birmingham, UK; ^6^Department of Nursing and Midwifery, Zahedan University of Medical Sciences, Zahedan, Iran

## Abstract

**Purpose:**

To present the youngest age ever reported for acute corneal hydrops with total corneal edema in a child with advanced bilateral keratoconus.

**Methods:**

Patient presentation in ophthalmic clinic. The patient underwent various clinical tests and examinations including anterior segment optical coherence tomography (AS-OCT) and Scheimpflug corneal tomography.

**Results:**

A 5-year-old girl presented with uncorrected distance visual acuity (UDVA) of 0.4 in the right eye and nonmeasurable UDVA associated with severe photophobia in her left eye of a 3-day duration. Intraocular pressure using the iCare tonometer was 14 and 5 mmHg in the right and left eyes, respectively. An old corneal hydrops scar and posterior subcapsular cataract (PSC) in the right eye and a total limbus to limbus corneal hydrops in the left eye were observed on slit-lamp examinations. Scheimpflug corneal tomography was possible in the right eye but, due to excessive irregularity and scaring, was not possible in the left eye. Corneal thinning and scarring were evident in the anterior segment optical coherence tomography in the right eye and very edematous cornea associated with stromal cleft and epithelial bullae in the left eye. A management plan consisting of topical hypertonic solution and ointment was started to reduce her symptoms.

**Conclusion:**

Acute corneal hydrops may be the presenting sign of keratoconus; however, extensive hydrops involving the total cornea area at a very young age is very rare and has not been previously reported in the literature.

## 1. Introduction

Corneal hydrops as a complication of keratoconus (KCN) is characterized by significant bullous edema of the cornea secondary to breaks in the Descemet membrane and an influx of aqueous into the stroma and epithelium of the cornea [1]. It has been postulated as a complication of eye-rubbing [2]. Various therapeutic options (medical or surgical interventions or conservative methods) were introduced to reduce patients' symptoms or shorten the healing period. These options include intracameral gas injection (air, sulfur hexafluoride (SF6) or perfluoropropane (C3F8)) to produce a gas tamponade or compression sutures such as pre-Descemet's membrane sutures [3–5]. Among the conservative approaches is the use of hypertonic saline eye drops/ointments or antiglaucoma agents, which helps to improve the condition by drawing fluid from the cornea or reducing the hydrodynamic pressure on the back surface of the cornea, respectively [6]. Corneal imaging techniques, such as anterior segment optical coherence tomography (AS-OCT), Scheimpflug tomography, and ultrasound biomicroscopy (UBM), provide both diagnostic and therapeutic guidelines. The advantages of AS-OCT are its noncontact property in delineating the location of intrastromal aqueous influx through the rupture in the Descemet layer and intrastromal clefts and its ease of use in young children [7, 8]. The current study presents the youngest age ever reported for an acute corneal hydrops with total corneal edema in a case with advanced bilateral keratoconus.

## 2. Case Presentation

A 5-year-old girl presented to the ophthalmic clinic after her parents noticed whitish discoloration in her left eye and the child exhibiting severe light sensitivity for a three-day duration. Her clinical history was negative for any systemic, congenital/genetic disorders, atopy (recurrent rhinitis and dermatitis), and/or chronic rubbing of the eye. There was no history of topical or systemic medications, and the family history was negative for any ectatic disease. Uncorrected distance visual acuity (UDVA) recorded as decimal notation was 0.4 in the right eye and not measurable in the left eye. Intraocular pressure measured using iCare tonometer (iCare IC100, iCare Finland, Oy, Finland) was 14 and 5 mmHg in the right and left eyes, respectively.

**Figure 1:**
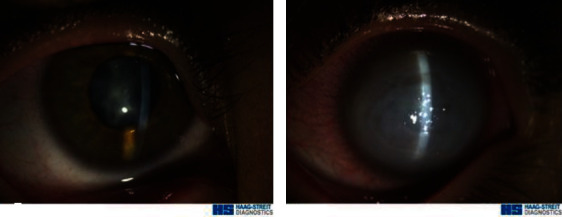
Slit-lamp photographs in the right (a) and left (b) eyes.

Slit-lamp biomicroscope examination showed an old corneal scar compatible with prior hydrops and a posterior subcapsular cataract (PSC) in the right eye and limbus to limbus corneal edema with hydrops in the left eye (Figure 1).

**Figure 2:**
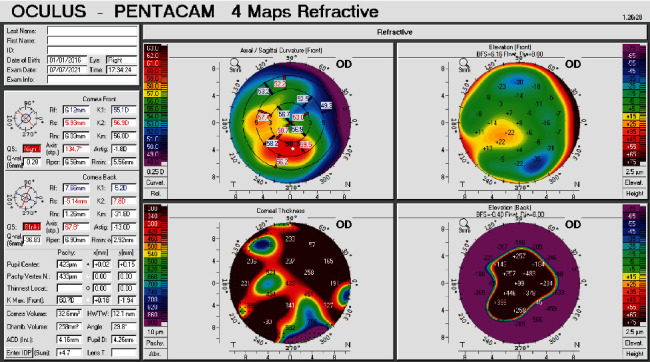
Scheimpflug image using Pentacam HR in the right eye.

Corneal topo/tomography was performed in the right eye, but due to excessive corneal irregularity secondary to the corneal scar, only a Scheimpflug image with an unacceptable quality specification (QS) was possible (Pentacam HR, Oculus; Wetzlar, Germany) after several attempts. A thin irregular cornea with a large posterior ectasia was noted on the corneal thickens and posterior elevation maps. The anterior elevation and anterior curvature showed milder changes with a maximum simulated keratometry of 60.7 diopters, possibly secondary to earlier scarring (Figure 2).

**Figure 3:**
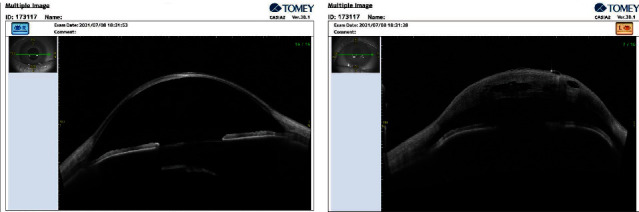
Anterior segment OCT in the right (a) and left (b) eyes.

AS-OCT of both eyes showed a thin and scarified cornea associated with an opacity under the posterior capsular surface in the right eye. The left eye had a very edematous cornea associated with multiple stromal clefts and epithelial bullae (Casia2 AS-OCT, Tomey Corporation, Nagoya, Japan) (Figure 3).

Dilated fundus examination was not possible but the attached retina was visible in both eyes on B-scan ultrasonography.

Based on the examination of prior hydrops in the right eye, a diagnosis of acute corneal hydrops was made in the left eye. Both the slit lamp exam and AS-OCT revealed edema extending to the limbus.

Her mother was informed about the nature and course of KCN and the possible future need for penetrating keratoplasty (PKP) in her left eye. Reliable autorefraction was not possible in the presence of corneal scar and PSC in the right eye. Due to severe photophobia, the patient did not cooperate enough to achieve accurate retinoscopic refraction in the right eye, and the decision was made to postpone for subsequent sessions after corneal hydrops reduction in the left eye. A management plan consisting of topical hypertonic solution and ointment was started, and it was emphasized that hydrops in most cases of KCN is a transient condition, and the patient will be followed for changes in her visual acuity, corneal transparency, and intraocular pressure.

## 3. Discussion

The classic view is that KCN begins as a progressive corneal disorder during puberty and progresses to midlife. KCN is less commonly seen in the very young, and this may be related to a delay in diagnosis or the inability of the very young to verbalize or note their visual complaints [9]. It was reported that the rate of disease progression, the frequency of more advanced KCN, and ultimately the need for corneal transplantation are greater in cases with early-onset [10]. Although KCN in children may be associated with systemic or genetic disorders such as Down syndrome and myotonic dystrophy; in otherwise healthy children, eye rubbing is considered a major predisposing factor for disease progression [11, 12].

The youngest child with KCN was reported in a 4-year-old girl secondary to vernal keratoconjunctivitis and sequentially eye rubbing without evidence of hydrops [11]. A 5-year-old boy with unilateral hydrops was previously reported. The corneal edema was limited to the temporal portion of the right cornea [13].

Although corneal hydrops can occur in various types of ectatic corneal disorders such as keratoconus, pellucid marginal degeneration (PMD), terrien marginal degeneration (TMD), and keratoglobus, it is often seen in association with keratoconus [14]. It occurs mostly in advanced stage of the disease and in younger patients, increasing the risk of visual impairment and the need for penetrating keratoplasty [15, 16].

Although with the introduction of corneal cross-linking as a treatment option for KCN by improving the mechanical strength of the cornea, the incidence of corneal hydrops appears to have decreased.

Several studies have reported corneal hydrops in children with KCN. A summary of the literature is provided in Table 1.

Gaskin and colleagues (2013) reported eye-rubbing, lower visual acuity at the first visit, and negative family history as risk factors for acute hydrops in KCN, while atopic disorders and a history of contact lens wear showed no association with hydrops. In their study, the average time to develop hydrops was 4 years following the detection of KCN [25]. In agreement with their study, our case had a negative family history of KCN. Although other studies found a higher prevalence of hydrops in severe allergic eye disease [16, 26], the mother of the present case denied chronic eye rubbing.

Often, severe and chronic rubbing of the eye is considered one of the main risk factor for hydrops, sometimes very minor trauma, such as the insertion of the contact lens has been reported as causative [26].

Multimodal imaging has improved the diagnosis of acute corneal hydrops associated with the assessment of corneal integrity. The AS-OCT of the present case showed structural changes in detail such as multiple stromal clefts and epithelial bullae as evident in the left eye delineated the location and extension of the Descemet's membrane break. Another application of AS-OCT is to determine the effectiveness of interventions such as intracameral gas injection in resolving acute edema. Basu et al.'s study showed that the measurement of break dimension (size and depth) in the DM correlates with the resolution of acute hydrops after intracameral injection of C3F8 [7]. Compared to UBM, which is a contact and operator-dependent method to assess DM integrity, AS-OCT is an easy, noncontact technique and more comfortable technique in children, capable of producing high-resolution images in conditions of corneal edema [27].

In conclusion, limbus to limbus involvement in corneal hydrops is in itself an uncommon presentation. To the authors' knowledge, this is the youngest reported case of corneal hydrops (5 years of age) associated with total corneal edema reported in the literature documented with AS-OCT.

## Figures and Tables

**Table 1:** Summary of several studies reporting corneal hydrops in children with KCN.

Study	Country	Age (years)/sex	Association
Rehany and Rumelt (1995) [13]	Israel	5/male	Bilateral advanced keratoconus, previous treatment for vernal conjunctivitis, and epithelial and stromal edema on the temporal side of the right cornea
Rahman and Anwar (2006) [17]	United Kingdom	6/female	Severe keratoconus
Rehany and Rumelt (1995) [13]	Israel	7/male	Advanced keratoconus with central stromal edema in the left eye with negative history of eye and systemic disorders and no history of habitual rubbing of the eye
Panahi-Bazaz et al. (2014) [18]	Iran	7/female	Bilateral corneal hydrops associated with eye rubbing secondary vernal keratoconjunctivitis (VKC)
Ioannidis et al. (2005) [19]	United Kingdom	7/female	Hydrops secondary to a chronic persistent eye-rubbing with no systemic involvement
Downie (2014) [20]	Australia	8/female	Bilateral acute corneal hydrops associated with severe atopy and no previous eye examination
Slim et al. (2015) [21]	Lebanon	10/male	Hydrops in an advanced KCN
Ozcan and Ersoz (2007) [22]	Turkey	11/male	Down syndrome and vigorous eye rubbing
Rehany and Rumelt (1995) [13]	Israel	11/male	Acute hydrops in the left eye in a bilateral advanced keratoconus, history of vernal conjunctivitis
Bilgin et al. (2013) [23]	Turkey	12/male	Simultaneous bilateral acute hydrops in Leber congenital amaurosis (LCA)
Khan et al. (2006) [24]	Saudi Arabia	16/male	Hydrops in a KCN patient with cornea plana
Current Study (2021)	Iran	5/female	Acute corneal hydrops associated with total limbus to limbus corneal edema, use of AS-OCT to enhance understanding of the structural corneal changes

## Data Availability

The patient's medical record is available.
